# DNA Polymerase Eta Participates in the Mutagenic Bypass of Adducts Induced by Benzo[a]pyrene Diol Epoxide in Mammalian Cells

**DOI:** 10.1371/journal.pone.0039596

**Published:** 2012-06-20

**Authors:** Alden C. Klarer, L. Jay Stallons, Tom J. Burke, Robert L. Skaggs, W. Glenn McGregor

**Affiliations:** 1 Department of Biochemistry and Molecular Biology, University of Louisville, Louisville, Kentucky, United States of America; 2 James Graham Brown Cancer Center, University of Louisville, Louisville, Kentucky, United States of America; 3 Department of Medicine, Division of Gastroenterology, Hepatology and Nutrition, University of Louisville, Louisville, Kentucky, United States of America; 4 Department of Pharmacology and Toxicology, University of Louisville, Louisville, Kentucky, United States of America; Louisiana State University and A & M College, United States of America

## Abstract

Y-family DNA-polymerases have larger active sites that can accommodate bulky DNA adducts allowing them to bypass these lesions during replication. One member, polymerase eta (pol eta), is specialized for the bypass of UV-induced thymidine-thymidine dimers, correctly inserting two adenines. Loss of pol eta function is the molecular basis for xeroderma pigmentosum (XP) variant where the accumulation of mutations results in a dramatic increase in UV-induced skin cancers. Less is known about the role of pol eta in the bypass of other DNA adducts. A commonly encountered DNA adduct is that caused by benzo[a]pyrene diol epoxide (BPDE), the ultimate carcinogenic metabolite of the environmental chemical benzo[a]pyrene. Here, treatment of pol eta-deficient fibroblasts from humans and mice with BPDE resulted in a significant decrease in *Hprt* gene mutations. These studies in mammalian cells support a number of *in vitro* reports that purified pol eta has error-prone activity on plasmids with site-directed BPDE adducts. Sequencing the *Hprt* gene from this work shows that the majority of mutations are G>T transversions. These data suggest that pol eta has error-prone activity when bypassing BPDE-adducts. Understanding the basis of environmental carcinogen-derived mutations may enable prevention strategies to reduce such mutations with the intent to reduce the number of environmentally relevant cancers.

## Introduction

Environmental mutagens such as the ultraviolet (UV) component of sunlight and chemical mutagens in food and cigarette smoke are well-established human carcinogens. One such compound is benzo[a]pyrene (B[a]P), which is a polycyclic aromatic hydrocarbon present in cigarette smoke, diesel exhaust and well-cooked meat. The carcinogenic effects of B[a]P are largely due to its metabolism to the highly reactive product benzo[a]pyrene diol epoxide (BPDE), which principally binds to the exocyclic amine of guanine [1]. Nucleotide excision repair recognizes and removes adducts induced by BPDE using transcription-coupled and global genomic repair mechanisms [2], [3]. However, if a cell enters S-phase with persistent damage, helix-distorting lesions halt the progression of the replication apparatus. Data indicate that specialized DNA polymerases that have relaxed base-pairing requirements may facilitate bypass of bulky DNA adducts in a process called translesion DNA synthesis (TLS), but with potentially mutagenic consequences. A subset of these proteins has been classified as Y-family polymerases based on structural similarities [4].

There are four members of the Y-family: REV1, pol eta, pol iota, and pol kappa. The role of these proteins in human disease is best characterized for pol eta. Loss of pol eta activity is the molecular defect underlying the XP variant syndrome [5], [6]. These patients have a very high incidence of sunlight-induced skin cancer but are nucleotide-excision repair proficient unlike the classic XP complementation groups A–G [7]. Cells from these patients are extremely hypermutable after exposure to UV due to the deficiency of pol eta which normally bypasses the most common UV-induced photoproducts, thymine-thymine cyclobutane pyrimidine dimers, in an error-free manner [8], [9]. In the absence of pol eta, the highly error-prone pol iota assumes this bypass function resulting in the accumulation of UV-induced mutations and an increased susceptibility to skin cancer [10]–[12].

The role of the Y-family proteins in the bypass of BPDE-induced adducts is considerably less clear. Pol kappa has been shown to participate in error-free bypass of lesions induced by BPDE *in vitro* using purified mouse and human enzymes [13]–[15]. Similar conclusions were reported using mouse cells lacking pol kappa activity [15], [16]. While there are some data concerning the role of pol eta in TLS of BPDE-induced DNA adducts, the data are somewhat contradictory. Purified pol eta has been shown to be capable of performing error-prone bypass of BPDE-adducted plasmids *in vitro* [13], [17], [18] and in mammalian cells using shuttle vectors to measure TLS efficiency and fidelity [19]. However, measurements of mutations at the endogenous hypoxanthine-guanine phosphoribosyltransferase (*HPRT)* locus in pol eta-deficient human cells treated with BPDE suggest that pol eta is error-free in bypassing BPDE-induced DNA adducts [20].

The B family member pol zeta is also implicated in the bypass of BPDE lesions. Purified pol zeta has alternatively been shown to have error-free [21] and error-prone [22], [23] bypass activity on BPDE-adducted DNA templates *in vitro*. In addition, pol zeta deficiency has been shown to affect TLS of BPDE lesions in mammalian cells [24], [25].

Here, BPDE treatment of both cells derived from pol eta-knockout mice and pol eta-deficient cells from an XP variant patient resulted in lower induced mutant frequencies. Our results suggest that pol eta is involved in error-prone bypass of BPDE-induced lesions and may be partially responsible for the mutagenic effects of this carcinogen. Understanding the mechanisms by which environmental chemicals induce mutations in DNA increases our understanding of how cancers can be linked to the environment and will increase our ability to develop strategies to prevent such cancers, for example through tissue-specific selective inhibition of one or more of these enzymes.

## Results

### Cytotoxicity from BPDE exposure is independent of pol eta status

**Figure 1:**
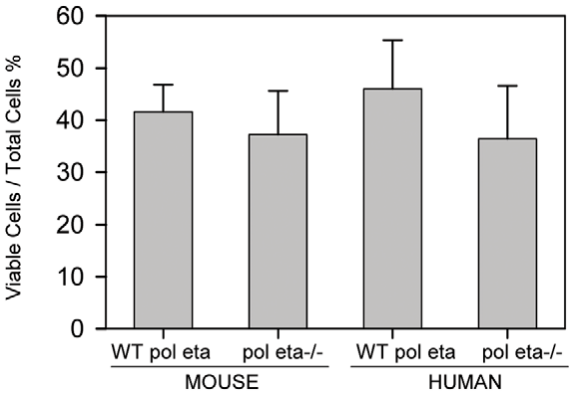
Polymerase eta does not affect cell survival after 150nM BPDE treatment. Viable cell calculations were made based on the number of clones present relative to the number of cells plated. This value was adjusted based on the number of clones observed in vehicle-only treated cells and expressed as average ± SEM.

The survival of mouse primary fibroblasts after treatment with 150 nM BPDE was assessed using clonogenic assays. Wild-type and pol eta knockout cells exhibited similar levels of cytotoxicity when exposed to 150 nM BPDE (41±5% and 37±8% survival, respectively, *p* = 0.67) (Figure 1). Human cells with wild-type pol eta and XP variant cells without functional pol eta exhibited similar sensitivities to 150 nM BPDE (46±9% and 36±10%, respectively, *p* = 0.51) (Figure 1). Mutagenic responses could therefore be assessed in comparable numbers of surviving cells after treatment.

### Reduced BPDE-induced mutant frequency was observed in the absence of pol eta

**Figure 2:**
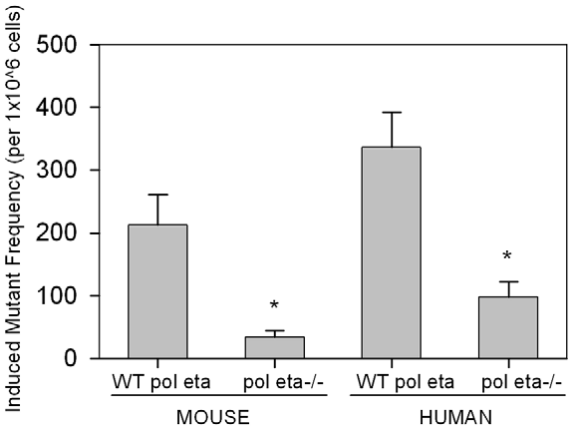
Induced mutant frequency is reduced in polymerase eta-deficient cells after BPDE treatment. Mutant frequency induced by 150 nM BPDE was calculated relative to the frequency of vehicle treated cells and expressed as average ± SEM. *p<0.05.

Fibroblasts deficient for pol eta were treated with 150 nM BPDE and mutant frequency was assessed by the formation of 6-thioguanine-(TG) resistant clones. The induced mutant frequency of wild type mouse fibroblasts in response to 150 nM BPDE was 213±48 mutants per 1×10^6^ cells. Relative to wild-type, the BPDE-induced mutant frequency in pol eta null mouse fibroblasts was significantly decreased, 34±10 mutants per 1×10^6^cells (*p*<0.05) (Figure 2).

Similarly, wild type human fibroblasts had a much higher induced mutant frequency relative to XP variant fibroblasts containing a non-functional pol eta. Normal human fibroblasts had an induced mutant frequency of 337±56 mutants per 1×10^6^cells while the induced mutant frequency in XP variant fibroblasts was 96±24 mutants per 1×10^6^cells (*p*<0.05) (Figure 2). Both human and mouse cells had low background mutation frequency which was not affected by the loss of pol eta (data not shown).

### Sequencing of Hprt mutant clones

After two weeks of growth in selective medium, macroscopic colonies of murine fibroblasts were isolated and lysed for RT-PCR amplification of *Hprt* cDNA and subsequent sequence analysis. Overall, the majority of the mutations in these mutant clones were guanine to thymine transversions which accounted for 50% and 70% of the mutations in wild-type and pol eta-null fibroblasts, respectively (Table 1).

**Table 1:** Kinds of Mutations Induced in the *Hprt* Gene of Murine Dermal Fibroblasts.

	Pol eta+/+		Pol eta−/−	
	No.	%	No.	%
Deletion of one Purine	1	4.55	1	4.35
Tandems	2	9.09	0	-
**Transitions**				
G•C → A•T	4	18.18	3	13.04
A•T → G•C	1	4.55	2	8.70
**Transversions**				
G•C → T•A	11	50.00	16	69.57
G•C → C•G	0	-	1	4.35
A•T → T•A	1	4.55	0	-
A•T → C•G	2	9.09	0	-
Total	22	100	23	100

## Discussion

The somatic mutation hypothesis of cancer asserts that changes arising from mutations in the genome can result in cells with a growth advantage and thus tumorigenic potential. While it is currently estimated that the spontaneous mutation rate in human cells is around 1–2×10^−8^ per nucleotide per generation [26], the overwhelming evidence for environmental carcinogen-induced cancers is attributed to the ability of these agents to cause DNA damage that results in the increased accumulation of mutations far exceeding those that occur spontaneously. The ability of a cell to bypass DNA-damage during replication prevents prolonged stalling of replication fork complexes that could signal cell death. However, depending on both the lesion encountered and the polymerase recruited, this bypass could result in replication errors that become mutations upon subsequent cell divisions. Polymerases of the Y-family are well known to participate in bypass replication and research has been focused on determining the accuracy of each enzyme when a particular DNA lesion is encountered. While polymerase kappa is likely specialized for protection against endogenously produced DNA adducts including those resulting from reactive oxygen species, this enzyme has also been implicated in the error-free bypass of BPDE lesions [13]–[15]. Other members of this polymerase family however, namely pol eta and pol iota, appear to have lower fidelity when they encounter BPDE-adducted bases. It was previously reported that pol eta may be involved in error-prone bypass of BPDE adducts using *in vitro* and yeast systems [18], [22], [27]. The only data examining pol eta in mammalian cells showed that human fibroblasts without a functional copy of pol eta were equally mutable relative to their wild-type counterparts after exposure to BPDE, indicating that pol eta does not participate in BPDE adduct bypass [20]. However, in this investigation, the eta-null XP variant cells and the normal human fibroblasts were not treated simultaneously, rather the normal fibroblast results were obtained from a different study in which a modified treatment protocol was used [28]. In the current study both murine and human fibroblasts without a functional pol eta accumulated fewer BPDE-induced mutations indicating an error-prone role for this enzyme in the bypass of BPDE-adducted DNA. Additionally, the current study used an asynchronous population of cells to determine mutant frequency while the previous studies used synchronized cells. Another possible reason for discrepancies in the induced mutant frequency in pol eta-deficient human cells between these two studies may be due to differences in the cell lines used as they were derived from different XP variant patients. XP4BE cells used by Watanabe *et* *al* [20] express a truncated version of pol eta that is 27 amino acids long due to a deletion of four nucleotides in the gene, while the XP115LO cells used in this study express a 127 amino acid truncated form as a result of a nonsense mutation. However, neither enzyme has been shown to have measurable enzyme activity. Data presented here from mouse cells support the low mutant frequency seen in human XP variant cells in a second species. In addition, pol eta was disrupted in these mice by removal of exon 4 and the generation of a frameshift mutation 30 bp downstream. The resultant mutant transcript would therefore encode a 92 amino acid truncated form of pol eta. This deletion is similar to that found in XP4BE cells and supports our hypothesis that loss of functional pol eta reduces BPDE-induced mutagenesis.

The observation of a reduced mutant frequency in pol eta-null mouse fibroblasts and in human fibroblasts deficient for pol eta relative to their pol eta wild-type counterparts in response to BPDE exposure indicates that this enzyme is involved in error-prone bypass of BPDE-adducts, in contrast to its error-free role in the bypass of UV-induced lesions. Thus, while it would be catastrophic to target pol eta in the body as a whole as an anti-cancer strategy, increasing the risk of UV-induced mutations and cancers, it may be possible to selectively inhibit this enzyme in tissues other than the skin that accumulate bulky lesions like those induced by BPDE. It was recently shown that ribozyme-mediated knockdown of the Y-family polymerase REV1 in the lung via aerosol delivery reduced the multiplicity of lung tumors in B[a]P-treated mice [29]. Lung-directed targeting of pol eta may be useful for those with increased exposure to environmental BPDE, as is the case for smokers. Determining the role of pol eta in the bypass of BPDE-adducts will further our knowledge of environmental carcinogen-linked cancers with the intent to determine possible prevention strategies.

## Materials and Methods

### Ethics Statement

This study was carried out in strict accordance with the recommendations in the Guide for the Care and Use of Laboratory Animals of the National Institutes of Health. The protocol was approved by the Institutional Animal Care and Use Committee at the University of Louisville protocol number 09059. All efforts were made to minimize animal suffering.

### Cells and Cell Culture

Pol eta knockout mice were a gift from the Kucherlapati laboratory [30]. These mice were backcrossed with C57BL/6 mice and were congenic in the C57BL/6 genetic background. Mice were tested for mutations in pol eta and pol iota using PCR-based protocols [31], [32]. All mice used were proficient for pol iota. Primary murine fibroblasts were established from 8- to 9- week-old mice using standard techniques as published [33]. Briefly, using an autoclaved ear punch, small pieces of ear from anesthetized mice were removed and plated in MEM-a (Life Technologies) supplemented with 10% fetal calf serum (Hyclone), 2mmol/L glutamine, nonessential amino acids (Life Technologies), penicillin (100 units/mL), streptomycin (100 ug/mL), and fungizone (Life Technologies; 1 ug/mL). Fibroblasts were grown at 37°C under hypoxic conditions (2–3% O2, 5% CO2) previously reported to increase the number of population doublings and lengthen the time before senescence of primary murine cells [34]. The medium was changed within 48 h, and the fungizone was omitted.

NF1604 human lung embryonic fibroblasts human fibroblasts were a gift from Dr. Lisa McDaniels [35] at the University of Texas Southwestern Medical Branch under the terms of an MTA between Greon Corporation and WGM. XP115LO fibroblasts were a gift from Dr. Veronica Maher [36]. Human fibroblasts were grown in DMEM (Gibco) supplemented with 10% supplemented calf serum (Atlanta Biologicals), penicillin (100 units/mL), and streptomycin (100ug/mL) (Sigma) at 37°C in 5% CO_2_.

### Exposure to BPDE

BPDE was purchased from the National Cancer Institute Chemical Carcinogen Repository. Powdered BPDE was dissolved in anhydrous tetrahydrofuran (THF) (Sigma). For cytotoxic and mutagenic studies the culture medium was aspirated and the cells were washed with sterile PBS (pH 7.4) and replaced with serum-free medium. BPDE was added to the culture medium to a final concentration of 150nM. The control cells were exposed to solvent (THF) only. After 1 hour incubation at 37°C, the cells were washed twice with PBS and the medium was replaced with complete medium.

### Cytotoxic and Mutagenic Assays

Primary mouse fibroblasts were assayed for cytotoxic and mutagenic responses to BPDE at early passages (six to eight population doublings) after primary cultures were established. A series of independent populations (1.5×10^6^ cells each, plated on 150-mm-diameter plates) were allowed to attach overnight before exposure to BPDE. Cytotoxic responses to BPDE were established by exposing the cells at the same density as used for the mutagenesis experiments, namely 1×10^4^ cells/cm^2^. After exposure, the cells were plated at cloning density to measure colony-forming ability. Fresh media was added 7 days after treatment and cells were stained with 1% crystal violet (Fisher Scientific) at 14 days. For mutagenesis, cells were allowed 3–5 days of growth before trypsinization and passage of 1.5×10^6^cells. After 8–9 days of post-treatment growth, cells were selected in 20uM 6-thioguanine (TG) at a density of 450 cells/cm^2^. Colony-forming ability was also determined at the time of TG selection by plating the cells at cloning density in nonselective medium and was used to correct for the mutant frequency. After 14–20 days, TG resistant clones were isolated for *Hprt* coding region amplification. After colony isolation, plates were stained, and mutant frequency was determined, defined as the number of observed TG clones per 10^6^ clonable cells (corrected for cloning efficiency). Data were analyzed using a student's t-test assuming unequal variance.

#### Amplification and Sequencing of *Hprt* cDNA

Isolation of thioguanine clones, reverse transcriptase-PCR of the *Hprt* cDNA, and sequence analysis of the PCR products were performed as previously described [10], [33].
